# Precarious work increases depression-based disability among male employees

**DOI:** 10.1093/eurpub/ckab119

**Published:** 2021-07-13

**Authors:** Pasi Pyöriä, Satu Ojala, Jouko Nätti

**Affiliations:** Faculty of Social Sciences, Tampere University, Tampere, Finland

## Abstract

**Background:**

Precarious employment is a potent occupational health risk, but little is known about its association with work-related disability and its causes. This study analyzes whether employment precariousness is associated with receiving disability pension (DP) due to depression and whether this differs according to gender.

**Methods:**

Statistics Finland’s Quality of Work Life Surveys (1997, 2003, 2008 and 2013) were merged with register-based DP data obtained from the Finnish Centre for Pensions. The survey material was used to measure employment precariousness using five variables: fear of job loss, poor employability prospects, previous unemployment, low earnings and temporary contracts. We followed 20–60-year-old employees until 2016 and studied Cox proportional hazard ratios (HRs) for receiving DP among women and men, adjusting for sociodemographic covariates, working conditions and health at baseline.

**Results:**

The overall risk of receiving DP tended to increase as precarious job features accumulated. Among men, a higher risk of receiving DP due to depression was associated with previous unemployment [HR 2.2, 95% confidence interval (CI) 1.1–4.2] and poor employability (HR 2.4, 95% CI 1.3–4.7), whereas no corresponding association was found among women.

**Conclusions:**

Employment precariousness may reflect a psychological stress mechanism that predisposes the individual to mental health problems, predicting future disability. Work disability risk shows gendered differences depending on the cause of DP. Promoting employability at workplace and policy levels could offset the health risks associated with precariousness.

## Introduction

Precarious work has been the subject of growing concern in recent decades.^1^ In the labor market context, precariousness means objective uncertainty in employment conditions (e.g. unemployment) and perceived job insecurity (e.g. fear of job loss). Stringent global competition, rapid technological changes and neoliberal economic policies are often seen as the forces that have paved the way for precariousness.^2–4^

In the labor market, precariousness represents a continuum of employment conditions that ranges from the secure full-time, well-compensated, and socially protected employment contract at one end, to a high degree of precariousness in different job features at the other.^5^^,^^6^ A precarious labor market position is characterized by, for example, periphery contracts, spells of unemployment, deteriorating working conditions, low income and lack of employment protection.^4^^,^^7–10^

In addition to objective uncertainty in employment, the scarring effects of perceived labor market risks play a key role in understanding precariousness and its consequences. Since Greenhalgh and Rosenblatt’s seminal work,^11^ which defined job insecurity as ‘the perceived powerlessness to maintain desired continuity in a threatened job situation’ (p. 438), subjective labor market risks have received a great deal of attention.

The implications of the fear of job loss for psychological distress and health are comparable in their severity to those of unemployment itself.^12^ Burgard et al.,^13^ for example, found that persistent perceived job insecurity is a significant predictor of poorer self-rated health. Otterbach and Sousa-Poza^14^ observed that not only being unemployed but also subjective job insecurity has a strong negative effect on both life satisfaction and health: the latter association being quite strong, up to half that of being unemployed.

The adverse effects of perceived job insecurity are, in many cases, exacerbated by poor employability prospects, i.e. the reduced capability of an individual to obtain new employment if required. Past adversities, such as experienced unemployment, may operate via lowered expectations of becoming unemployed in the future, and fear of the future is destructive to a person’s subjective well-being.^15^ Loss of control over one’s ‘destiny’ is at the heart of this vicious circle, with potentially detrimental consequences for health and work ability.

Green^16^ emphasizes that employability strongly moderates the effects of unemployment and job insecurity. Employability matters not only for the unemployed but also for employees whose well-being and life satisfaction may depend on the perceived probability of being able to attain another job that is as good as their current one. According to Green, employability is particularly important for men: an increase in men’s employability from zero to 100% reduced the detrimental effect (measured by self-rated life satisfaction and mental health) of job insecurity by more than half: Even where there was no job insecurity, more employable individuals had greater life satisfaction, although there was no significant effect on mental health in this circumstance.

In this study, we analyse the association between precarious labor market position and the risk of receiving disability pension (DP), and ask how the accumulation of precariousness predicts DP.^10^^,^^17^ Our study concerns Finnish pay earners aged 20–60, and focuses on gender differences. Although research on precarious work has grown rapidly in recent years, its relation to granted DPs remains less understood, especially from the gender perspective.

We base our analysis on accounts according to which the possible detrimental effects of job insecurity on mental health might be greater for men than for women.^16^ We focus on depression-based disability because it is the leading cause for granting DP in Finland. Earlier studies on cause-specific DP have paid little attention to gender, the focus being on physical work exposures,^18^ job strain,^19^ relational justice^20^ and worktime.^21^ Among these studies, only Vahtera et al.^21^ addressed the gender aspect: Self-assessed worktime control decreased the risk of receiving DP based on mental disorders among women.

Conceptually, we maintain that accumulating uncertainty, i.e. precarious labor market position, measured by five validated indicators,^17^ is an independent determinant of DP. We hypothesize that precariousness, especially poor employability,^16^ predicts future disability. We also hypothesize that precariousness differentiates the risk of receiving DP between women and men, in particular DP based on the depression. This is one of the first studies to assess the gendered contours of precariousness in relation to the cause-specific risk of receiving DP.

## Methods

### Study population

The study comprised 15 338 employees aged 20–60 (8142 women and 7196 men) who had been interviewed for Statistics Finland’s Quality of Work Life Surveys in 1997 (*N* = 2873), 2003 (*N* = 3922), 2008 (*N* = 4119) or 2013 (*N* = 4424). These surveys were based on random samples, representing 15–64-year-old wage- and salary-earners residing in Finland. The samples are drawn from the official Labor Force Surveys that comply with the EU regulation.

The cross-sectional survey material was pooled and linked to a follow-up extending until the end of 2016, which contained register-based information on all DP recipients. The DP data, using the employees’ encrypted personal identification codes, were obtained from the Finnish Centre for Pensions. The survey material’s missing data problem was only marginal, because the material was collected through face-to-face interviews by trained interviewees. The response rates in all the surveys were high: 69–79%. The merged administrative registers contain annual measurements for all these respondents with no further missing data. Only migration to another country or death terminate the cumulation of the follow-up data.

### Measures

#### Disability pension

The outcome variable comprised all cases receiving a work-related DP, including the number of people drawing a full-time or partial DP annually at the end of each follow-up year. DP recipients may participate in occupational training/rehabilitation. As a methodological limitation, we can consider only first DPs during the follow-up. Those who were granted DP during the baseline survey year were excluded from the analysis.

Granted DPs due to mental and physical diagnoses were classified according to the ICD-10 criteria. Depression (F32–F33) and mental disorders (other F-code diagnoses), followed by musculoskeletal diseases (M-code diagnoses), are the leading causes for granting DP in Finland. Diagnoses based on depression and other mental disorders cover over one-third of all new DPs in the country, with depression being the main single cause for DP retirement. Over the period of the present study, about 20 000–25 000 people retired on earnings-related DP annually.

In Finland, a DP may be granted (either for a fixed term or until further notice) to an insured person aged 16–64 whose work ability has been reduced for at least one year due to an illness, injury or handicap. In recent years, about one-tenth of full disability pensioners and about 80% of partial disability pensioners have been engaged in paid work. Retirees who have received DP due to mental diagnoses work less frequently than those who have received DP due to musculoskeletal diseases or other diseases.^22^

The risk of DP receipt increases with age, and the tendency to retire on a DP is highest around the age of 60. This varies by the cause: those retiring due to mental diagnoses are younger than those retiring due to musculoskeletal diseases. People of lower socioeconomic status run a higher risk of disability retirement than people of upper socioeconomic status; however, socioeconomic differences due to depression are small.^23^

#### Indicators of precariousness

Based on earlier research hypothesizing that precariousness acts as a stressor to the individual, predisposing to work-related disability,^10^^,^^17^ we used the following measures that reflect both subjective and objective precariousness: fear of job loss, poor employability prospects, previous unemployment, low earnings and temporary contracts.

Fear of job loss was the sum of three risk factors: the perceived threat of being laid off, dismissed and/or made redundant, formed as a dichotomous variable (no threats vs. at least one threat). Poor employability was measured by asking ‘What do you think would be the likelihood of you finding a new job: good, reasonable or poor?’ The response was deemed to indicate precariousness if the respondent felt they had poor chances of finding a new job in the open labor market. Previous unemployment referred to at least one spell of unemployment in the past five years. By low earnings, based on the question ‘What is your monthly gross pay in your main job before tax?’, we refer to the lowest income quintile. Finally, our multi-dimensional construct of precariousness included employment with a fixed-term contract.

#### Covariates

Sociodemographic covariates included age, gender and educational level. Prior evidence indicates that the dropout rate from the labor force and the proportion of time spent on DP increase as people get older.^24^ In Finland, women spend more time on DP due to mental disorders and musculoskeletal diseases than men, and people with primary or secondary education spend more time on DP than people with tertiary education in all disease categories.^24^

We included measures for intimate relationship (married or cohabiting vs. no relationship) and having children under 18 living at home (Yes/No). Intimate relationship and family provide social support that is associated with positive health outcomes and psychological well-being.^25^ As chronic illnesses (mental health problems in particular) co-exist with work disability,^26^ we controlled for long-term morbidity by including a measure for reported chronic illnesses (Yes/No) or depressive symptoms (Weekly or daily/Less often). We also controlled for the survey year in all models.

The covariates characterizing working conditions included adverse physical factors, job demands/control and shiftwork, as these job characteristics have been linked to disability.^18^^,^^21^^,^^22^ Physical work exposures were based on an 18-item inventory. Following prior studies,^18^ we used a factor analysis that divided the items into three factors: hazardous, physical workload and ‘office work’ exposures.

The first factor comprised the following items: heat, cold, vibration, draught, noise, smoke, gases, fumes, humidity, dusts, dirtiness of the work environment, poor/glaring lighting and irritant substances. The second factor comprised items related to repetitive and monotonous movements, difficult working positions and heavy lifting. The third factor combined items somewhat different from ‘computer work’, used by Halonen et al.,^18^ interpretable however as office-related work, describing inadequate ventilation, restlessness of the work environment, lack of space and mildew in buildings. These covariates were not categorized, in order to accurately measure the variation in the individuals’ responses in the different survey years.

We also adjusted for several items that are very similar to those in previous studies utilizing the Job Demand-Control (JDC) model.^27^ High job demands and/or low autonomy have been associated with an increased risk of receiving DP, whereas good work control implies the opposite.^28^

Job demands were measured using six items that reflect adverse work environment factors: Time pressure and tight time schedules: Very much = 1…Not at all = 5; Over the past few years, do you think your pace of work has: Increased considerably = 1…Decreased considerably = 5; In your work, can you generally take breaks or rest periods: Sufficiently often, Not quite often enough, Far too seldom; My work contains tight time schedules: Totally true = 1…Totally untrue = 4; I do not have time to do my work as well and conscientiously as I would like to: Totally true = 1…Totally untrue = 4; There are too few employees for the workload at my workplace: Totally agree = 1…Totally disagree = 5.

Job control (autonomy) was measured using five items: Are you able to influence: The contents of your tasks; The order in which you do your tasks; The pace of your work; Your working methods; The divisions of tasks between employees (1 = Not at all…4 = A great deal). The JDC measures were first summed and then divided into quintiles.

Finally, we included a variable for shiftwork.^18^ The dichotomized variable included two- and three-shift work with or without night work. Those in day work, morning- or evening work only, as well as ‘total working time’ contracts, were categorized as day work.

### Statistical analyses

We tested the covariates for multicollinearity. In the follow-up model, we computed Cox proportional hazard ratios (HRs) for receiving DP, adjusting for the covariates. We estimated the associations between gender and receiving DP, and interacted gender with precariousness (table 2), to validate further analysis that separates genders.

In the first analysis, indicators on precariousness were analysed separately in relation to the risk of receiving DP for any cause and depression (table 3). We applied a four-step procedure considering precarious labor market position only (model 1), demographic/socio-economic characteristics (m2), working conditions (m3), and finally, to study the potential endogeneity of the precarious population (pre-existing mental/physical illnesses determining DP rather than precariousness), baseline depression and long-term illness (m4). In the second analysis, following prior studies^10^^,^^17^^,^^29^ recommending the use of multidimensional exposures, we combined the indicators to assess how the accumulation of precariousness predicts future disability (table 4). The analyses were separated by gender, as we expected that the mechanisms behind work disability are different for women and men.^16^^,^^17^^,^^21^ The results were broken down by all causes of DP and depression, as we found that certain precarious job features were more strongly and consistently related to the risk of receiving DP due to depression than other diagnoses.

All survey respondents were included in the analysis, with follow-up running from the baseline year (1997, 2003, 2008, 2013) up until 2016. Those who died/moved out of the country (*n* = 410 men, *n* = 538 women) were not excluded from the analysis, because Cox regression considers the censored structure of the data. We also experimented with fixed-length follow-ups (3 years; 5 and 8 years without the 2013 survey), and with the duration of unemployment during the follow-up, to check the robustness of our results. The associations remained robust, with minor differences in the magnitude of HRs.

## Results

Table 1 shows the characteristics of the study population by all DP cases and depression-based DP, including the distribution of precarious job features and all covariates. The supplementary online table presents these characteristics by gender. Table 2 validates the gender difference in entering the DP scheme as regards exposure to precariousness.

**Table 1:** Baseline covariates and their associations with all-cause disability pension (DP) and DP based on depression

	Participants				DP: All causes			DP: Depression		
Covariate	***N***		**%**		***N***		**%**	***N***	**%**	
All participants	15 338		100.0		939		6.1	135		0.9
Gender										
Man	7196		46.9		401		5.6	47		0.7
Woman	8142		53.1		538		6.6	88		1.1
Precarious features (Exposure)										
Fear of job loss										
Yes	4409		28.8		294		6.7	45		1.0
No	10 926		71.2		645		5.9	90		0.8
Poor employability										
Yes	4192		27.4		461		11.0	49		1.2
No	11 135		72.6		476		4.3	86		0.8
Temporary contract										
Yes	2104		13.7		132		6.3	18		0.9
No	13 230		86.3		807		6.1	117		0.9
Lowest pay quintile										
Yes (NB categorized variables 15–20% per survey yr)	2644		17.2		207		7.8	28		1.1
No	12 694		82.8		732		5.8	107		0.8
Previous unemployment										
Yes	3730		24.3		251		6.7	35		0.9
No	11 605		75.7		687		5.9	99		0.9
Demographic and socioeconomic factors (model 2)										
Age at the baseline										
20–30	3098		20.2		57		1.8	25		0.8
31–40	3941		25.7		122		3.1	35		0.9
41–50	4503		29.4		420		9.3	57		1.3
51–60	3796		24.7		340		9.0	18		0.5
In a relationship (baseline)										
Yes	10 663		69.5		637		6.0	83		0.8
No	4675		30.5		302		6.5	52		1.1
Child <18 yrs in the household (baseline)										
Yes	6545		42.7		332		5.1	66		1.0
No	8793		57.3		607		6.9	69		0.8
Education										
Primary	2181		14.2		249		11.4	22		1.0
Secondary	7213		47.0		506		7.0	62		0.9
Tertiary	5944		38.8		184		3.1	51		0.9
Working conditions (model 3)										
Shift work										
Yes	3190		20.8		225		7.1	29		0.9
No	12 141		79.2		712		5.9	106		0.9

	**Mean (StD)**		**StD**		**Mean (StD)**		**StD**	**Mean**		**StD**

Hazardous, scale 1…5 (No … High exposure)	1.5		0.6		1.7		0.7	1.6		0.7
Physical work load, scale 1…5 (Low … High exposure)	1.7		1.0		2.0		1.1	1.9		1.1
Office work, scale 1…5 (Low … High exposure)	1.5		0.7		1.6		0.8	1.7		0.8
Demands, scale 1…4 (Low … High demands)	2.5		1.1		2.7		1.2	2.7		1.2
Control, scale 1…4 (Low … High control)	2.5		1.1		2.3		1.1	2.3		1.1
Baseline physical and mental illnesses (model 4)										
Baseline depressive symptoms Weekly/Daily										
Yes	335		2.2		59		17.6	19		5.7
No	15003		97.8		880		5.9	116		0.8
Baseline longterm illness										
Yes	4726		30.8		536		11.3	72		1.5
No	10605		69.2		403		3.8	63		0.6

**Table 2:** Hazard ratios (HR) and their 95% confidence intervals for gender (*1), and for interaction terms precarious job features X gender (*2), derived from Cox proportional hazard models

	DP: All causes		DP: Depression	
	HR	95% CI	HR	95% CI
HR for gender (*1)				
Woman (ref. man)	1.10	0.97–1.25	1.65	1.15–2.35
HR for precarious features X gender (^*^2)				
Woman (ref. man)	1.36	1.10–1.79	3.01	1.70–5.33
Fear of job loss	1.12	0.90–1.40	1.42	0.76–2.63
Fear of job loss X woman	0.96	0.71–1.30	0.96	0.44–2.01
Poor employability	1.80	1.46–2.21	2.82	1.53–5.18
Poor employability X woman	0.70	0.54–0.91	0.41	0.20–0.86
Temporary contract	1.17	0.83–1.64	0.49	0.17–1.47
Temporary contract X woman	0.85	0.54–1.32	1.63	0.45–5.84
Lowest pay quintile	1.48	1.09–2.01	1.30	0.56–3.03
Lowest pay quintile X woman	0.96	0.67–1.39	0.84	0.32–2.25
Previous unemployment	1.46	1.15–1.86	1.90	1.01–3.59
Previous unemployment X woman	0.70	0.50–0.99	0.33	0.14–0.80

* 1 Adjusted for survey yr (1997, 2003, 2008, 2013) and age.

* 2 Adjusted for survey yr. age and other precarious features of the job.

Statistically significant HRs are bolded.

Tables 1 and 2 indicate that a slightly higher proportion of women had entered the DP scheme due to any cause and depression than men. The interaction term in table 2 shows, however, that in comparison to all employees who received DP, men exposed to poor employability prospects and previous experience of unemployment had entered both all-cause DP and depression-based DP more probably than women.

As shown in table 3, precariousness was found to increase the risk of receiving DP due to any cause with varying degrees. In the model 1, men who feared losing their jobs were at an elevated risk of receiving DP, but after adjusting for the covariates, this insecurity factor became statistically insignificant. In the model 1, poor employability and low pay appeared to be the most significant risk factors for receiving DP for both genders. However, when all covariates were adjusted for, poor employability remained a significant predictor of receiving DP only among men. Also, previous unemployment predicted disability for men (all covariates adjusted for in model 4).

**Table 3:** Hazard ratios (HRs) by all causes of DP and DP based on depression, by exposure to precarious job features

Precarious job feature			DP: All causes			DP: Depression		
			**Cases/Exposed**	**HRs**	**CI**	**Cases/Exposed**	**HRs**	**CI**
Fear of job loss	Models 1	Men	138/2079	1.38	1.12–1.69	18/2079	1.65	0.91–2.98
		Women	156/2330	1.15	0.96–1.39	27/2330	1.15	0.73–1.82
	Models 2	Men		1.36	1.10–1.67		1.66	0.92–3.01
		Women		1.12	0.93–1.36		1.16	0.73–1.83
	Models 3	Men		1.05	0.83–1.32		1.23	0.65–2.33
		Women		1.00	0.82–1.24		1.24	0.76–2.04
	Models 4	Men		1.02	0.81–1.28		1.08	0.56–2.06
		Women		0.97	0.79–1.10		1.15	0.70–1.88
Poor employability	Models 1	Men	190/1661	1.88	1.52–2.33	20/1661	3.05	1.62–5.76
		Women	271/2531	1.33	1.11–1.61	29/2531	1.28	0.78–2.11
	Models 2	Men		1.65	1.33–2.05		3.06	1.61–5.82
		Women		1.23	1.02–1.49		1.30	0.79–2.15
	Models 3	Men		1.56	1.25–1.95		2.74	1.43–5.25
		Women		1.21	1.00–1.46		1.25	0.75–2.07
	Models 4	Men		1.45	1.16–1.82		2.42	1.25–4.69
		Women		1.09	0.90–1.32		1.07	0.64–1.79
Temporary contract	Models 1	Men	50/701	1.73	1.28–2.35	4/701	0.84	0.30–2.39
		Women	82/1403	1.17	0.92–1.49	14/1403	0.80	0.44–1.46
	Models 2	Men		1.71	1.26–2.31		0.83	0.29–2.38
		Women		1.15	0.90–1.46		0.77	0.42–1.42
	Models 3	Men		1.34	0.94–1.89		0.50	0.16–1.50
		Women		1.11	0.83–1.50		0.80	0.41–1.59
	Models 4	Men		1.25	0.88–1.77		0.45	0.15–1.35
		Women		1.13	0.84–1.53		0.81	0.41–1.62
Lowest pay quartile	Models 1	Men	54/749	1.97	1.47–2.65	7/749	1.80	0.77–4.19
		Women	153/1895	1.54	1.27–1.86	21/1895	1.10	0.66–1.81
	Models 2	Men		1.57	1.17–2.12		1.79	0.76–4.23
		Women		1.30	1.07–1.58		1.06	0.63–1.79
	Models 3	Men		1.30	0.95–1.77		1.53	0.63–3.72
		Women		1.28	1.04–1.57		1.13	0.66–1.94
	Models 4	Men		1.27	0.93–1.74		1.49	0.61–3.63
		Women		1.23	1.00–1.51		1.03	0.60–1.77
Previous unemployment	Models 1	Men	128/1829	1.68	1.35–2.08	19/1829	2.07	1.13–3.78
		Women	123/1901	1.18	0.96–1.45	16/1901	0.68	0.39–1.18
	Models 2	Men		1.50	1.20–1.86		2.14	1.16–3.95
		Women		1.11	0.90–1.36		0.66	0.38–1.15
	Models 3	Men		1.22	0.95–1.57		2.03	1.05–3.92
		Women		1.04	0.81–1.33		0.64	0.35–1.18
	Models 4	Men		1.29	1.01–1.65		2.16	1.12–4.18
		Women		1.03	0.80–1.32		0.63	0.34–1.16

Model 1: Adjusted for survey yr (1997, 2003, 2008, 2013) and age.

Model 2: + Demographic and socioeconomic covariates.

Model 3: + Other precarious features and working conditions.

Model 4: + Baseline physical and mental illnesses (full model).

After adjusting for the covariates, poor employability (men’s HR 2.4, 95% CI 1.3–4.7) and previous unemployment (men’s HR 2.2, 95% CI 1.1–4.2) were the only precarious job features that elevated the risk of receiving DP due to depression. This finding applied to only men (table 3).

Table 4 presents the accumulation of precariousness in relation to the risk of entering the DP scheme. In our model for the accumulation, with all covariates adjusted for (model 4), the HRs for receiving DP due to any cause among men who were exposed to one or more job insecurity factors and were at the level of 1.5–1.8 (95% CI 1.2–2.4). There was a tendency for the risk of receiving DP to increase as precarious job features accumulated.

**Table 4:** Hazard ratios (HRs) for causes of DP, by exposure to the accumulated precariousness of the job (scale: summed 0…5 items, not weighted, categorized 0, 1, 2–5)

		DP: All causes			DP: Depression		
		**Cases/Exposed**	**HRs**	**CI**	**Cases/Exposed**	**HRs**	**CI**
Model 1	Men, 0 precarious features	93/2864	Ref.		11/2864	Ref.	
	1	155/2477	1.79	1.38–2.33	15/2477	1.74	0.79–3.79
	2–5	153/1843	2.53	1.95–3.30	21/1843	3.44	1.63–7.27
	Women. 0 precarious features	127/2575			29/2575		
	1	190/2792	1.19	0.95–1.50	32/2792	1.10	0.66–1.84
	2–5	218/2766	1.58	1.26–1.98	26/2766	0.88	0.51–1.51
Model 2	Men. 0 precarious features						
	1		1.61	1.24–2.08		1.73	0.79–3.80
	2–5		2.17	1.66–2.83		3.60	1.69–7.69
	Women. 0 precarious features						
	1		1.09	0.87–1.38		1.09	0.65–1.82
	2–5		1.37	1.09–1.71		0.86	0.49–1.49
Model 3	Men. 0 precarious features						
	1		1.53	1.18–1.99		1.61	0.73–3.54
	2–5		1.90	1.45–2.49		3.18	1.46–6.90
	Women. 0 precarious features						
	1		1.10	0.87–1.38		1.07	0.64–1.80
	2–5		1.35	1.07–1.69		0.81	0.46–1.41
Model 4	Men. 0 precarious features						
	1		1.52	1.17–1.97		1.59	0.73–3.51
	2–5		1.79	1.36–2.35		2.70	1.23–5.91
	Women. 0 precarious features						
	1		1.03	0.82–1.30		0.97	0.58–1.62
	2–5		1.24	0.98–1.56		0.66	0.38–1.16

Model 1: Adjusted for survey yr (1997, 2003, 2008, 2013) and age.

Model 2: + Demographic and socioeconomic covariates.

Model 3: + Working conditions.

Model 4: + Baseline physical and mental illnesses (full model).

In our model for the accumulation, with all covariates adjusted for, men who suffered from 2–5 simultaneous precarious job features were at an elevated risk of receiving DP due to depression (HRs 2.7, 95% CI 1.2–5.9). We found no such risk for women. Overall, we found that the men who were exposed to precariousness in our survey population were at risk of entering the DP scheme due to depression.

## Discussion

In this study, poor employability prospects and previous unemployment elevated the risk of receiving DP due to depression among men, but we found no corresponding association among women, supporting our hypothesis that precarious labor market situation predicts future disability and affects women and men differently. The disadvantage that men had in this regard was statistically significant even after controlling for key sociodemographic background variables, baseline depression and long-term illness, and various factors related to working conditions. We also found that the accumulation of precariousness is more harmful than a single threat. The accumulation of uncertainty increased the risk of receiving DP due to depression among men but, again, not among women.

Prior research on the gendered contours of precarious work and its association with work disability has produced mixed results. Discrepancies between study outcomes reflect variations in research settings and differences in national labor markets. In particular, the lack of cause-specific data on DP recipients may hamper understanding the gendered mechanisms between different work environment exposures and DP diagnoses.

In Spain, Vives et al.^30^ observed an association between precarious work and poor mental health, which was stronger among women, suggesting an interaction with gender-related power asymmetries. Ojala and Pyöriä,^17^ on the other hand, found that Finnish men whose labor market position reflected precariousness and who had experienced unemployment in the past were at a higher risk of entering the DP scheme than women in a similar position. Ferrante et al.,^31^ in the Italian context, found that precarious work was not directly associated with poor mental health, but was related to economic problems, possibly caused by job instability. Their finding only applied to men. Park et al.^32^ estimated how depression among older people in Korea varied by employment status and gender: The risk of depression in male precarious workers was significantly increased compared with full-time male permanent workers, whereas it was not significantly different among women.

The changing economic landscape may play a role in feelings of uncertainty among the study population at risk of receiving DP. In Finland, men’s level of education is lower than that of women, and men are typically employed in cyclical industries such as manufacturing.^33^ Men employed in manufacturing may have more industry-specific skills than (female) service workers, whose jobs involve interactive and social skills. As Green^16^ has proposed, job loss (hence also job insecurity) has a greater effect on individuals who possess fewer transferable skills and are hence less employable.

Prior research has shown that the labor market history, especially prolonged unemployment, predicts work disability.^34^ The detrimental effects of unemployment and the fear of job loss might be greater for men than for women.^16^ Job insecurity may pose a threat to men’s masculine self-identity, which revolves around paid work and the breadwinner status.^35^ Unemployment is possibly more stigmatizing and harmful to men than it is to women, who benefit from more inclusive social networks and social support outside the workplace. Sociability plays a protective role against depression and may postpone retirement, whereas weak social ties may be a risk factor for mental health problems, especially as job insecurity increases.^36^^,^^37^

The main strength of the present study is that the survey material reliably represented all pay earners of the country and had very high response rates. A further strength is the high-quality register-based data on granted cause-specific DPs as the outcome. We were also able to control for several important sociodemographic and job-related background factors. However, the survey material enabled us to measure the exposure to precariousness at baseline only. The survey also lacked information on the respondents’ health behavior risks, but we were able to study the probable endogeneity of precarious workers by controlling long-term illnesses and depressive symptoms. Unfortunately, our data had no information on vocational rehabilitation. Another limitation is that a marginal share of the shortest spells of DP may be uncovered since the follow-up indicator (main status) is only based on one’s status at the end of a year.

Finally, our statistical model predicting HRs might overestimate the risk for receiving DP since we did not consider the competing risks of labor market exit. However, as we focused on 20–60-year-old employees, the DP system is the main route of labor market exit. In the study population, possible competing exit routes such as part-time pension only concern older population.

## Conclusions

Depression is widespread across Europe. In addition to developing mental health policies and clinical practices, the promotion of job quality should be given a high priority in order to make work more sustainable. Attention should also be paid to amending perceived labor market risks. A positive attitude toward job opportunities is an important coping mechanism for an individual, whereas cynicism about the future often has the opposite effect. The risk of receiving DP could potentially be offset by improving individuals’ employability through developing skills, autonomy and opportunities for on-the-job learning.

## Supplementary data

Supplementary data are available at *EURPUB* online.

## Supplementary Material

ckab119_Supplementary_Data
