# Supplementary material for: Precarious work increases depression-based disability among male employees
Source: Eur J Public Health. 2021 Jul 13;31(6):1223–30. doi: 10.1093/eurpub/ckab119 (PMC8643409; doi:10.1093/eurpub/ckab119)
Supplement: ckab119_Supplementary_Data [file ckab119_supplementary_data.docx]

**Online supplementary table.** Baseline covariates and their associations with all-cause disability pension (DP) and DP based on depression, by gender.

|  | Participants, women | | Participants, men | | DP: All causes, women | | DP: All causes, men | | DP, women: Depression | | DP, men: Depression | |
| --- | --- | --- | --- | --- | --- | --- | --- | --- | --- | --- | --- | --- |
| Covariate | N | % | N | % | N | % |  |  | N | % | N | % |
| All participants | 8142 | 100.0 | 7193 | 100.0 | 538 | 6.6 | 401 | 5.6 | 88 | 1.1 | 47 | 0.7 |
|  |  |  |  |  |  |  |  |  |  |  |  |  |
| Precarious features (Exposure) | | | | | | | | | | | | |
| Fear of job loss |  |  |  |  |  |  |  |  |  |  |  |  |
| Yes | 2330 | 28.6 | 2079 | 28.9 | 156 | 6.7 | 138 | 6.6 | 27 | 1.2 | 18 | 0.9 |
| No | 5812 | 71.4 | 5114 | 71.1 | 382 | 6.6 | 263 | 5.1 | 61 | 1.0 | 29 | 0.6 |
| Poor employability |  |  |  |  |  |  |  |  |  |  |  |  |
| Yes | 2531 | 27.4 | 1661 | 23.1 | 271 | 10.7 | 190 | 11.4 | 29 | 1.1 | 20 | 1.2 |
| No | 5605 | 72.6 | 5530 | 76.9 | 265 | 4.7 | 211 | 3.8 | 59 | 1.1 | 27 | 0.5 |
| Temporary contract |  |  |  |  |  |  |  |  |  |  |  |  |
| Yes | 1403 | 17.2 | 701 | 9.7 | 82 | 5.8 | 50 | 7.1 | 14 | 1.0 | 4 | 0.6 |
| No | 6738 | 82.8 | 6492 | 90.3 | 456 | 6.8 | 351 | 5.4 | 74 | 1.1 | 43 | 0.7 |
| Lowest pay quintile |  |  |  |  |  |  |  |  |  |  |  |  |
| Yes (NB categorized variables 15–20% per survey yr) | 1895 | 23.3 | 749 | 10.4 | 153 | 8.1 | 54 | 7.2 | 21 | 1.1 | 7 | 0.9 |
| No | 6247 | 76.7 | 6447 | 89.6 | 385 | 6.2 | 347 | 5.4 | 67 | 1.1 | 40 | 0.6 |
| Previous unemployment |  |  |  |  |  |  |  |  |  |  |  |  |
| Yes | 1901 | 23.4 | 1829 | 25.4 | 123 | 6.5 | 128 | 7.0 | 16 | 0.8 | 19 | 1.0 |
| No | 6239 | 76.6 | 5366 | 74.6 | 414 | 6.6 | 273 | 5.1 | 71 | 1.1 | 28 | 0.5 |
| Demographic and socioeconomic factors (model 2) | | | | | | | | | | | | |
| Age at the baseline |  |  |  |  |  |  |  |  |  |  |  |  |
| 20–30 | 1534 | 18.8 | 1564 | 21.7 | 28 | 1.8 | 29 | 1.9 | 16 | 1.0 | 9 | 0.6 |
| 31–40 | 1970 | 24.2 | 1971 | 27.4 | 70 | 3.6 | 52 | 2.6 | 23 | 1.2 | 12 | 0.6 |
| 41–50 | 2481 | 30.5 | 2022 | 28.1 | 235 | 9.5 | 185 | 9.1 | 36 | 1.5 | 21 | 1.0 |
| 51–60 | 2157 | 26.5 | 1639 | 22.8 | 205 | 9.5 | 135 | 8.2 | 13 | 0.6 | 5 | 0.3 |
| In a relationship (baseline) |  |  |  |  |  |  |  |  |  |  |  |  |
| Yes | 5633 | 69.2 | 5030 | 69.9 | 360 | 6.4 | 277 | 5.5 | 52 | 0.9 | 31 | 0.6 |
| No | 2509 | 30.8 | 2166 | 30.1 | 178 | 7.1 | 124 | 5.7 | 36 | 1.4 | 16 | 0.7 |
| Child <18 yrs in the household (baseline) |  |  |  |  |  |  |  |  |  |  |  |  |
| Yes | 3425 | 42.1 | 3120 | 43.4 | 196 | 5.7 | 136 | 4.4 | 46 | 1.3 | 20 | 0.6 |
| No | 4717 | 57.9 | 4076 | 56.6 | 342 | 7.3 | 265 | 6.5 | 42 | 0.9 | 27 | 0.7 |
| Education |  |  |  |  |  |  |  |  |  |  |  |  |
| Primary | 1027 | 12.6 | 1154 | 16.0 | 119 | 11.6 | 130 | 11.3 | 10 | 1.0 | 12 | 1.0 |
| Secondary | 3539 | 43.5 | 3674 | 51.1 | 292 | 8.3 | 214 | 5.8 | 44 | 1.2 | 18 | 0.5 |
| Tertiary | 3576 | 43.9 | 2368 | 32.9 | 127 | 3.6 | 57 | 2.4 | 34 | 1.0 | 17 | 0.7 |
| Working conditions (model 3) | | | | | | | | | | | | |
| Shift work |  |  |  |  |  |  |  |  |  |  |  |  |
| Yes | 1866 | 22.9 | 1324 | 18.4 | 150 | 8.0 | 75 | 5.7 | 21 | 1.1 | 8 | 0.6 |
| No | 6274 | 77.1 | 5867 | 81.6 | 387 | 6.2 | 325 | 5.5 | 67 | 1.1 | 39 | 0.7 |
|  | Mean | StD |  |  | Mean | StD | Mean | StD | Mean | StD | Mean | StD |
| Hazardous, scale 1…5 (No … High exposure) | 1.4 | 0.5 | 1.7 | 0.7 | 1.5 | 0.5 | 1.9 | 0.8 | 1.5 | 0.6 | 1.8 | 1.0 |
| Physical workload, scale 1...5 (Low … High exposure) | 1.8 | 1.0 | 1.7 | 1.0 | 2.1 | 1.1 | 2.0 | 1.1 | 1.9 | 0.8 | 2.0 | 1.3 |
| Office work, scale 1...5 (Low … High exposure) | 1.6 | 0.7 | 1.5 | 0.6 | 1.7 | 0.8 | 1.5 | 0.7 | 1.8 | 0.8 | 1.6 | 0.6 |
| Demands, scale 1…4 (Low … High demands) | 2.7 | 1.1 | 2.4 | 1.1 | 2.8 | 1.1 | 2.5 | 1.1 | 2.6 | 1.2 | 2.8 | 1.0 |
| Control, scale 1…4 (Low … High control) | 2.4 | 1.1 | 2.6 | 1.1 | 2.3 | 1.1 | 2.4 | 1.1 | 2.3 | 1.1 | 2.6 | 1.1 |
| Baseline physical and mental illnesses (model 4) | | | | | | | | | | | | |
| Baseline depressive symptoms Weekly/Daily |  |  |  |  |  |  |  |  |  |  |  |  |
| Yes | 215 | 2.6 | 120 | 1.7 | 38 | 17.7 | 21 | 17.5 | 13 | 6.0 | 6 | 5.0 |
| No | 7927 | 97.4 | 7076 | 98.3 | 500 | 6.3 | 380 | 5.4 | 75 | 0.9 | 41 | 0.6 |
| Baseline long-term illness |  |  |  |  |  |  |  |  |  |  |  |  |
| Yes | 2588 | 31.8 | 2138 | 29.7 | 323 | 12.5 | 213 | 10.0 | 47 | 1.8 | 25 | 1.2 |
| No | 5550 | 68.2 | 5055 | 70.3 | 215 | 3.9 | 188 | 3.7 | 41 | 0.7 | 22 | 0.4 |
